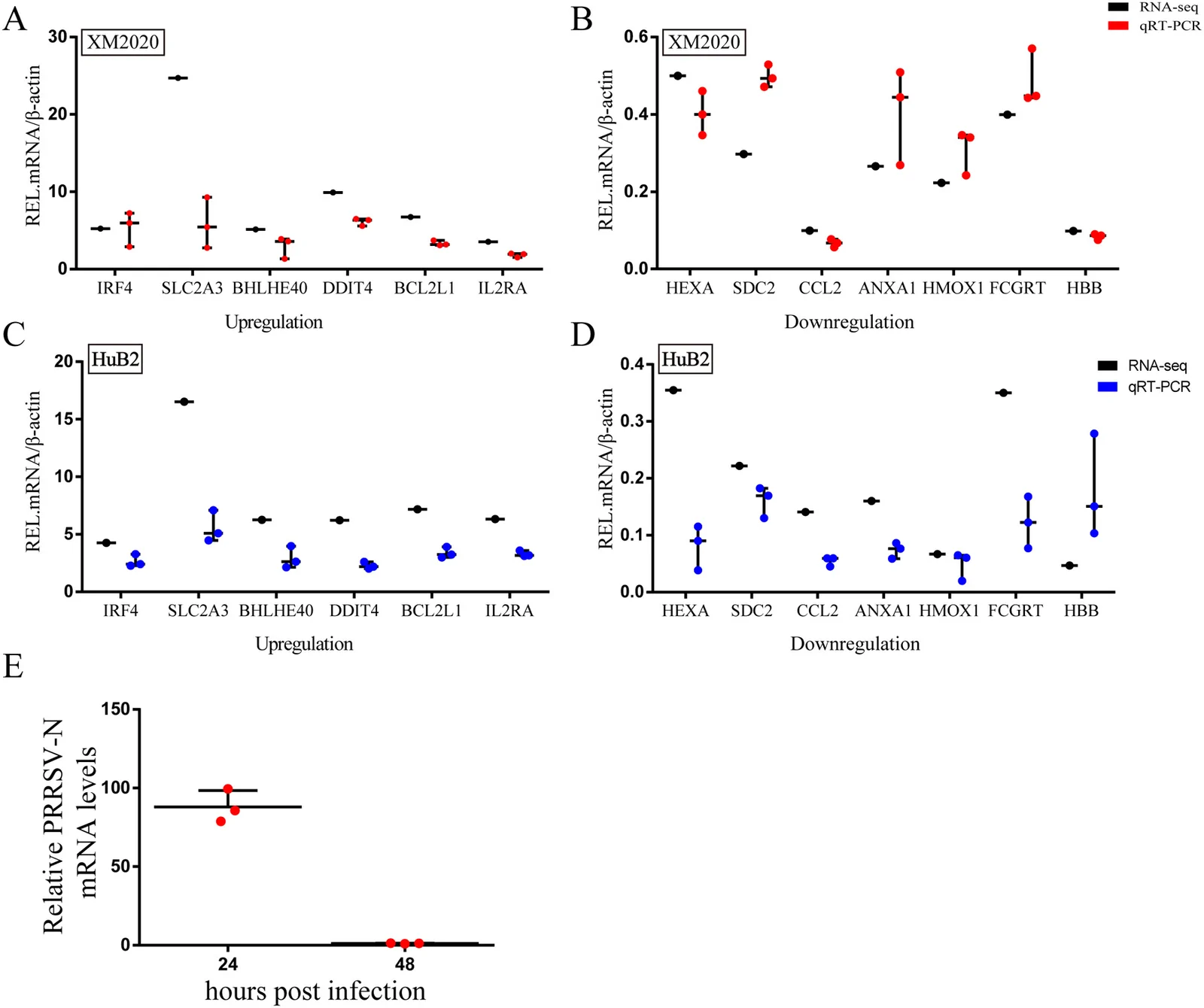# Protein M of NADC30-like PRRSV induces MARCH6-mediated K33-linked polyubiquitination of SLA-DRA

**DOI:** 10.1016/j.jbc.2026.113478

**Published:** 2026-08-25

**Authors:** Ruochen Hu, Sujing Mou, Chenfei Yu, Jinyao Li, Wenli Shi, Yining Zhang, Saba Nasir, Ruhai Guo, Junda Li, Xinxin Qiu, Shuqi Xiao, Xinglong Wang

**Affiliations:** 1 College of Veterinary Medicine, Northwest A&F University, Yangling, China; 2 Department of Animal Engineering/Shaanxi Engineering Research Center of the Prevention and Control for Animal Disease, Shaanxi Vocational and Technical University of Agriculture and Forestry, Yangling, Shaanxi Province, China; 3 State Key Laboratory for Animal Disease Control and Prevention, Lanzhou Veterinary Research Institute, Chinese Academy of Agricultural Sciences, College of Veterinary Medicine, Lanzhou University, Lanzhou, Gansu, China

**Keywords:** NADC30-like PRRSV, MHC molecules, E3 ubiquitin ligase, immune evasion, antigen presentation

## Abstract

NADC30-like porcine reproductive and respiratory syndrome virus (PRRSV) has replaced the high-pathogenicity PRRSV (HP-PRRSV) as the predominant circulating strain in China. Unlike HP-PRRSV, NADC30-like PRRSV exhibits increased genetic diversity and enhanced persistence for persistent infection, along with altered host immune responses, which collectively complicate disease control. In this study, we found that infection with the NADC30-like representative strain XM2020 induced a weaker antibody response than infection with the HP-PRRSV strain HuB2, and that SLA-DRA expression was downregulated. *In vitro* analyses further demonstrated that this suppression of SLA-DR in peripheral blood mononuclear cells is independently of viral replication. Mechanistically, XM2020-M binds to SLA-DRA and recruits the E3 ubiquitin ligase MARCH6, promoting K33-linked polyubiquitination and proteasomal degradation of SLA-DRA, a function not observed for HuB2-M. In addition, XM2020-M stabilizes MARCH6 by inhibiting its degradation, thereby enhancing its ubiquitin ligase activity. Further mapping identified residue 93 of XM2020-M as a critical determinant of this process. Collectively, these findings reveal a novel immune evasion mechanism by which XM2020-M impairs antigen presentation by targeting the degradation of SLA-DRA, which may contribute to altered adaptive immune responses and viral persistence associated with NADC30-like strains.

Porcine reproductive and respiratory syndrome virus (PRRSV), a member of the Arteriviridae (order *Nidovirales*) family of RNA viruses, shares taxonomy with related viruses such as simian hemorrhagic fever virus (SHFV), equine arteritis virus (EAV), and lactate dehydrogenase-elevating virus (LDV) (1, 2). Since its appearance in China in 1996, PRRSV has experienced two main epidemiological shifts: the dominance of highly pathogenic PRRSV (HP-PRRSV) from 2006 (3), followed by NADC30-like strains overtaking from 2013 (4, 5). NADC30-like PRRSV strains have a high recombination rate (6, 7). In China, this contributes to their rich genetic diversity and significant variation in pathogenicity, with virulence typically falling between that of the parental strains (8). Compared with HP-PRRSV and classical PRRSV (C-PRRSV), NADC30-like strains harbor multiple amino acid mutations in the ORF5 and nsp2 regions (9), which are concentrated in neutralizing epitopes, potential N-glycosylation sites, and signal peptide regions, and may be involved in regulating viral virulence, entry, replication, and immune evasion (10). Currently, although modified-live virus (MLV) and inactivated vaccines are available, the cocirculation of NADC30-like strains, and PRRSV modified-live virus vaccines in some swine farms poses challenges for effective disease control (8), suggesting that NADC30-like PRRSV may employ immune evasion mechanisms distinct from those of HP-PRRSV.

PRRSV uses multiple strategies to evade host immunity. This allows persistent infection and delays adaptive immune responses. Effective adaptive immunity depends on antigen presentation by antigen-presenting cells (APCs). In pigs, this process relies on swine leukocyte antigen (SLA) molecules, which are essential for the activation of CD4^+^ T cells and subsequent antibody production, as well as for CD8^+^ T cell-mediated cellular immunity. Previous studies show PRRSV can impair antigen presentation by downregulating β2-microglobulin and SLA class I molecules, thereby affecting CD8^+^ T cell responses (11). However, the regulation of SLA-II expression during PRRSV infection remains controversial (12, 13, 14). Notably, some NADC30-like strains do not significantly reduce CD4^+^ T cell in piglets, yet it still fails to elicit strong neutralizing antibodies (15). This suggests these viruses may disrupt SLA-II–mediated antigen presentation, rather than directly depleting T cells. Therefore, elucidating how PRRSV, particularly NADC30-like strains, modulates SLA-II expression is critical for understanding impaired humoral immunity during infection.

Various pathogens exploit diverse mechanisms to modulate MHC molecules, including transcriptional suppression, post translational modification, and manipulation of host signaling pathways. For example, the BDLF3 protein of Epstein-Barr virus (EBV) mediates ubiquitination and subsequent proteasomal degradation of MHC I/II molecules (16); the Zta protein from Epstein-Barr virus suppresses CIITA transcription, thereby downregulating MHC II expression (17); and Kaposi's sarcoma-associated herpesvirus (KSHV) employs an indirect mechanism to modulate human leukocyte antigen-DRA degradation by utilizing MARCH8 as the targeting factor (18). These multifaceted strategies underscore the sophisticated immune evasion mechanisms evolved by viruses. Notably, PRRSV has also evolved immune evasion mechanisms targeting MHC class II molecules.

In this study, we compared early antibody responses after infection with HP-PRRSV and NADC30-like PRRSV. We then investigated how NADC30-like strains downregulate SLA-DRA (a swine MHC class II molecule). Our results show that the M protein of NADC30-like strains recruits the E3 ubiquitin ligase MARCH6. This leads to K33-linked polyubiquitination of SLA-DRA, thereby significantly reducing its expression. These findings reveal how PRRSV establishes immunosuppression by interfering with antigen presentation. They also help explain why NADC30-like strains have gradually become predominant in clinical settings.

## Results

### NADC30-like PRRSV exhibits enhanced replication and suppresses SLA-DRA expression in piglets

To investigate the differences in viral replication dynamics and humoral immune responses between NADC30-like and HP-PRRSV infections, we conducted a comparative infection experiment in piglets. We used the NADC30-like strain XM2020 and the HP-PRRSV strain HuB2, monitoring replication and immune responses over time. As shown in Figure 1*A*, PRRSV-naïve piglets were inoculated with these strains, and peripheral blood samples were collected at 3-, 5-, 7-, and 14-days post infection (dpi) after each challenge. After viral titers peaked, XM2020 maintained slightly higher viral loads than HuB2 (Fig. 1*B*). *In vitro* assays further supported this finding: XM2020 replicated more efficiently than HuB2 (Fig. S1, *A* and *B*). Consistent with these results, PRRSV N protein-specific antibodies first appeared at 14 dpi in XM2020-infected piglets and then increased. However, anti-N protein antibody levels in XM2020 remained consistently lower than in HuB2-infected piglets (Fig. 1*C*). Given the key role of SLA-DR in antigen presentation and antibody production (19, 20), we next measured SLA-DRA/DRB transcription in peripheral blood *via* quantitative PCR (qPCR). XM2020 infection initially suppressed SLA-DRA transcription. During secondary (14 dpi) and tertiary (28 dpi) infections, its expression returned to baseline but did not increase (Fig. 1*D*). In contrast, HuB2 also suppressed SLA-DRA transcription during primary infection, but expression gradually increased during later infections (Fig. 1*D*). For SLA-DRB, early infection with either strain showed no significant inhibition (Fig. 1*E*). At the late stage, HuB2 infection significantly increased SLA-DRB expression, whereas XM2020 kept expression at baseline (Fig. 1*E*). Overall, these results suggest the weaker humoral immune response from XM2020 infection likely results from SLA-DRA downregulation.

**Figure 1:**
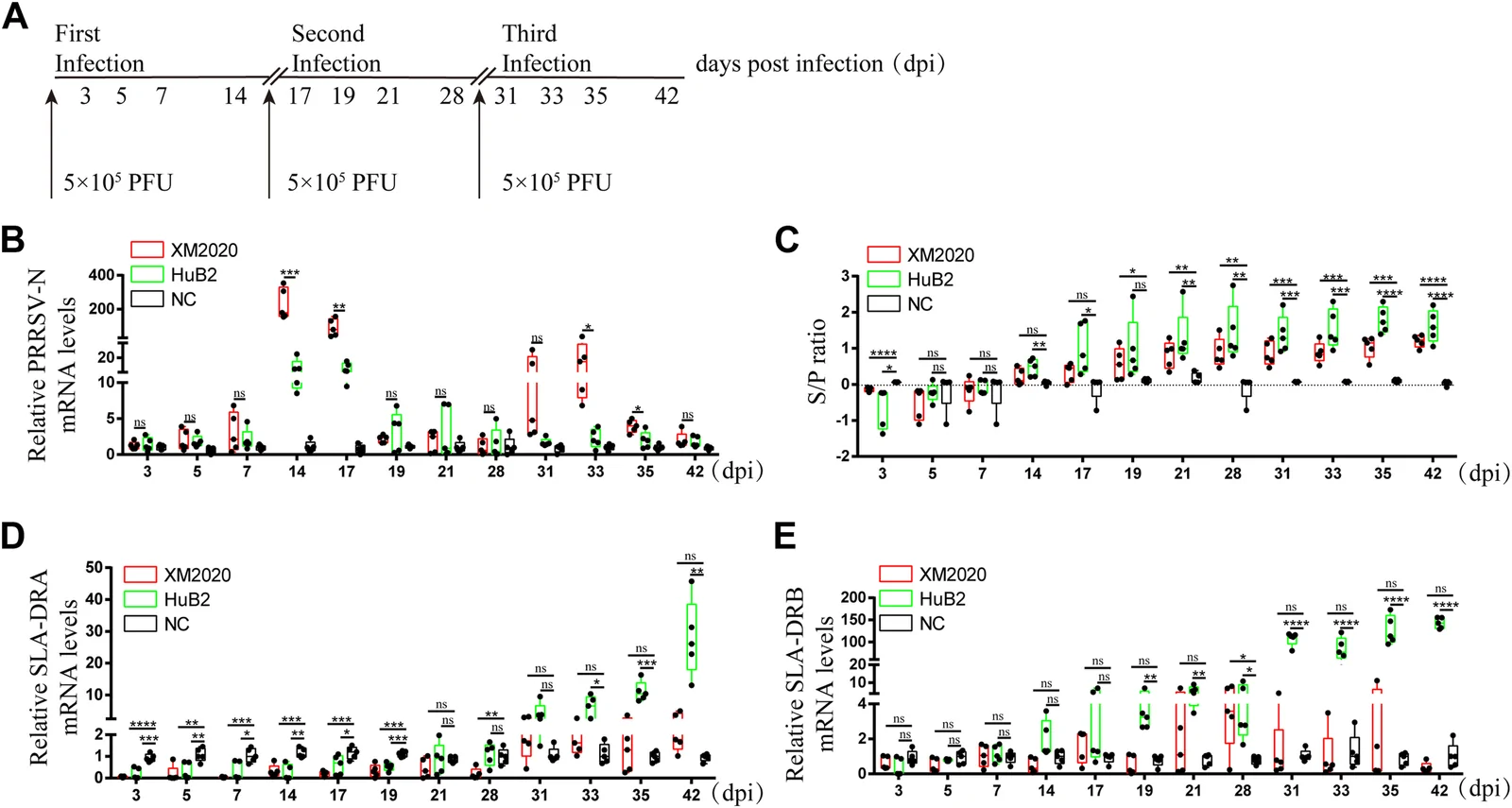
**Antibody response in XM2020 infection compared to HuB2 infection.***A*, PRRSV-naïve pigs were infected with XM2020 or HuB2 strain. Samples were collected from the peripheral blood of each pig at specified days post infection and analyzed accordingly. *B*, the levels of PRRSV-N transcripts in peripheral blood were measured at various days post infection using quantitative PCR (qPCR). Data are presented as mean values ± SDs (n = 5 pigs per group). Statistical significance was determined using two-tailed, unpaired Student’s t-tests. Not significant (ns); ∗*p* < 0.05, ∗∗*p* < 0.01, ∗∗∗*p* < 0.001. *C*, to assess the production of PRRSV N protein-specific antibodies, peripheral blood was obtained at the indicated days post infection. Sera were tested using a VDPro PRRSV AB ELISA kit. Mean values ± SDs are shown (n = 5 pigs per group), and statistical significance was assessed as above: ns, not significant; ∗*p* < 0.05, ∗∗*p* < 0.01, ∗∗∗*p* < 0.001, ∗∗∗∗*p* < 0.0001. *D*-*E*, quantitative analyses of SLA-DRA and SLA-DRB transcripts were performed on peripheral blood collected on various days after infection. Transcript levels were assessed by qPCR. Mean values ± SDs (n = 5 pigs per group) are shown, and significance was tested as above: ns, not significant; ∗*p* < 0.05, ∗∗*p* < 0.01, ∗∗∗*p* < 0.001, ∗∗∗∗*p* < 0.0001. PRRSV, porcine reproductive and respiratory syndrome virus; qPCR, quantitative PCR; SD, standard deviation; SLA, swine leukocyte antigen.

### NADC30-like PRRSV suppresses SLA-DRA expression in a replication-independent manner

As described above, XM2020 infection downregulates SLA-DRA and SLA-DRB transcription in peripheral blood. To further examine how XM2020 regulates antigen presentation by porcine peripheral blood mononuclear cells (PBMCs), we isolated PBMCs from piglets after three PRRSV infections, treated them with XM2020 or HuB2 *in vitro*, and performed transcriptome sequencing. XM2020 upregulated costimulatory molecules (CD80/CD86) and several SLA class I genes (SLA-3, -5, -6, -7, -8), whereas reducing SLA class II gene transcription, including SLA-DR and -DM (Fig. 2*A*). qPCR confirmed the transcriptomic results (Fig. S2, *A*–*D*). However, expression and regulation of SLA-II molecules in APCs after PRRSV infection remain controversial (12, 13, 21, 22). To address this, we further characterized SLA-DR regulation by infecting PBMCs with XM2020 or HuB2 *in vitro*. Western blot showed that XM2020 and HuB2 reduced SLA-DR protein levels in PBMCs (Fig. 2*B*). However, qPCR showed that XM2020 significantly downregulated both SLA-DRA and SLA-DRB, while HuB2 had minimal effects (Fig. 2*C*). After further integration of transcriptomic data with the in *vitro* and in *vivo* results, the downregulation of SLA-DRB by XM2020 appeared less consistent across different experimental conditions. Specifically, although SLA-DRB transcription was downregulated in the in *vitro* PBMC infection model, its transcript level in the peripheral blood of infected piglets remained unchanged from baseline. Thus, the downregulation of SLA-DRB by XM2020 was not consistently observed across all experimental settings. Given that PBMCs are not direct target cells of PRRSV (Fig. S2*E*) (23), we next examined if this regulation depends on viral replication. To this end, we infected PBMCs with either live or UV-inactivated XM2020, and found that UV inactivation did not abolish the ability of XM2020 to modulate SLA-DR expression (Fig. 2*D*). In addition, UV-inactivated XM2020 also downregulated SLA-DR expression in porcine alveolar macrophages (PAMs) (Fig. 2*E*). Collectively, these results indicate that XM2020 suppresses SLA-DR expression in PBMCs in a replication-independent manner (24).

**Figure 2:**
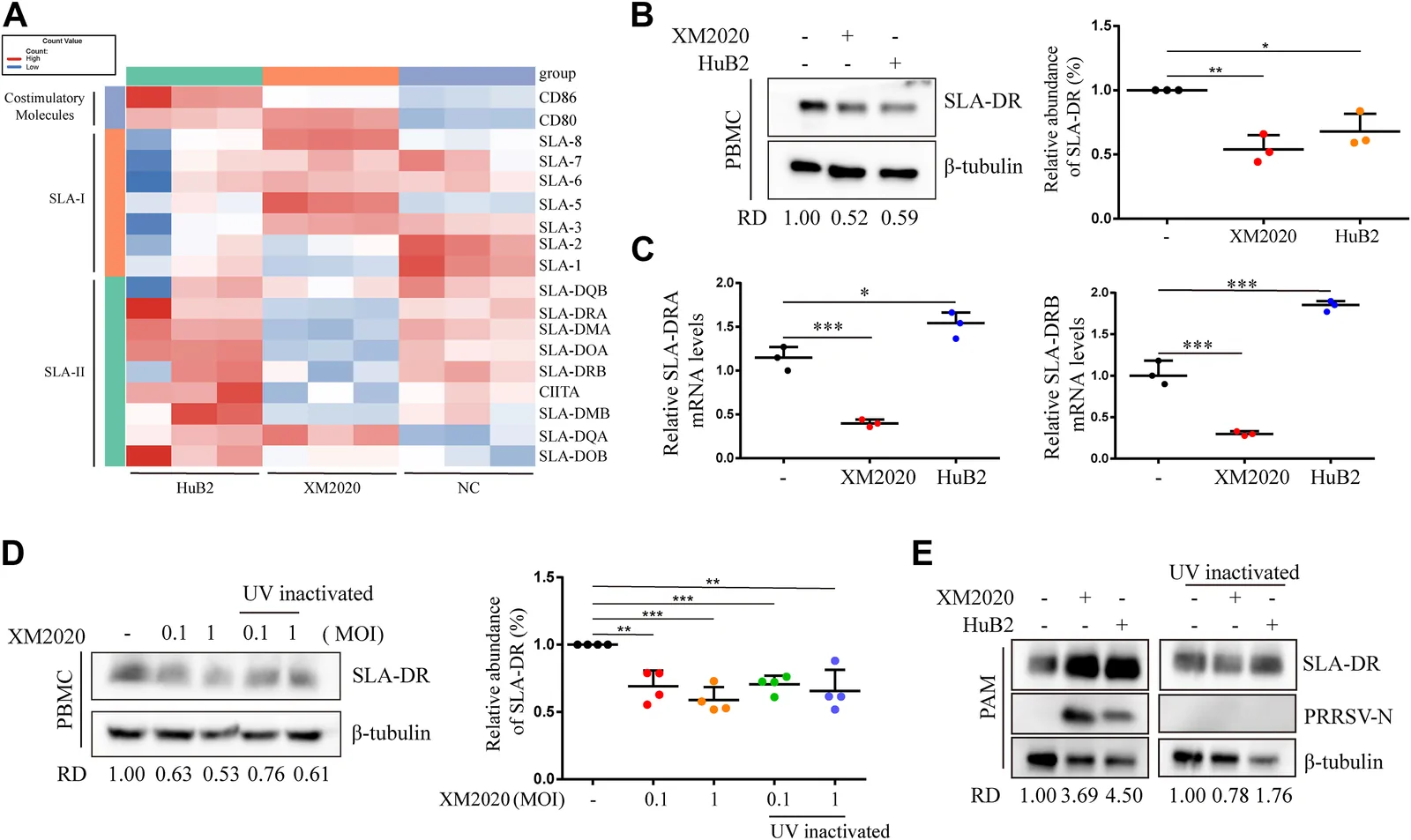
**XM2020 downregulates the SLA-DR expression in XM2020-convalescent pigs PBMCs, which are independent of viral replication.***A*, PBMCs from XM2020-convalescent pigs were treated with PRRSV (XM2020 or HuB2) at a multiplicity of infection (MOI) 1 for 24 h and then harvested for RNA-seq analysis. mRNA expression patterns of costimulatory molecules (CD80/CD86) and SLA molecules (heat map). *B*, XM2020-convalescent pig PBMCs were treated with XM2020 or HuB2 at 1 MOI, and SLA-DR protein levels were detected by western blot at the indicated time points. Representative blots from independent experiments using different donor PBMCs are shown. Because baseline SLA-DR expression varied between donors, all quantifications were performed relative to the within-blot control, with densitometric analysis shown on the left. Data are presented as mean ± SD (n = 3). Significance was assessed using two-tailed unpaired Student's t-tests. ∗*p* < 0.05, ∗∗*p* < 0.01. *C*, XM2020-convalescent pig PBMCs were treated with XM2020 or HuB2 at 1 MOI, and SLA-DRA and SLA-DRB mRNA levels were detected by qPCR at the indicated time points. Data are presented as mean ± SD (n = 3). Significance was assessed using two-tailed unpaired Student's t-tests. ∗*p* < 0.05, ∗∗∗*p* < 0.001. *D*, PBMCs from XM2020-convalescent pigs were treated with live or UV-inactivated XM2020 (MOI=0.1, 1) for 24 h, and samples were collected to analyze SLA-DR protein expression. See note in panel (*B*) for details regarding baseline variation. *E*, PAMs were infected with live or UV-inactivated XM2020 (MOI=0.1, 1) for 24 h, and samples were collected to analyze SLA-DR protein expression. PAM, porcine alveolar macrophage; PBMC, peripheral blood mononuclear cell; PRRSV, porcine reproductive and respiratory syndrome virus; qPCR, quantitative PCR; SLA, swine leukocyte antigen.

### NADC30-like PRRSV downregulated SLA-DRA expression

To identify the viral determinant for XM2020-mediated, replication-independent SLA-DR downregulation in PBMCs, we screened PRRSV structural proteins. HEK-293T cells were co-transfected with GFP-tagged structural proteins from XM2020 or HuB2 and MYC-tagged SLA-DRA and SLA-DRB. Western blot showed several structural proteins reduced SLA-DRA expression. XM2020-M markedly decreased SLA-DRA, whereas the reducing effect of HuB2-M was relatively weak (Fig. 3, *A* and *B*). These results suggesting that the M protein may account for the differential regulation of SLA-DRA between these two strains. None of the structural proteins from either strain affected SLA-DRB (Fig. 3, *C* and *D*), indicating specificity to SLA-DRA. Immunofluorescence showed XM2020-M colocalized with SLA-DRA in the cytoplasm, while HuB2-M showed limited co-localization (Fig. 3*E*). Co-immunoprecipitation (Co-IP) assays confirmed that XM2020-M directly interacts with SLA-DRA (Fig. 3*F*).

**Figure 3:**
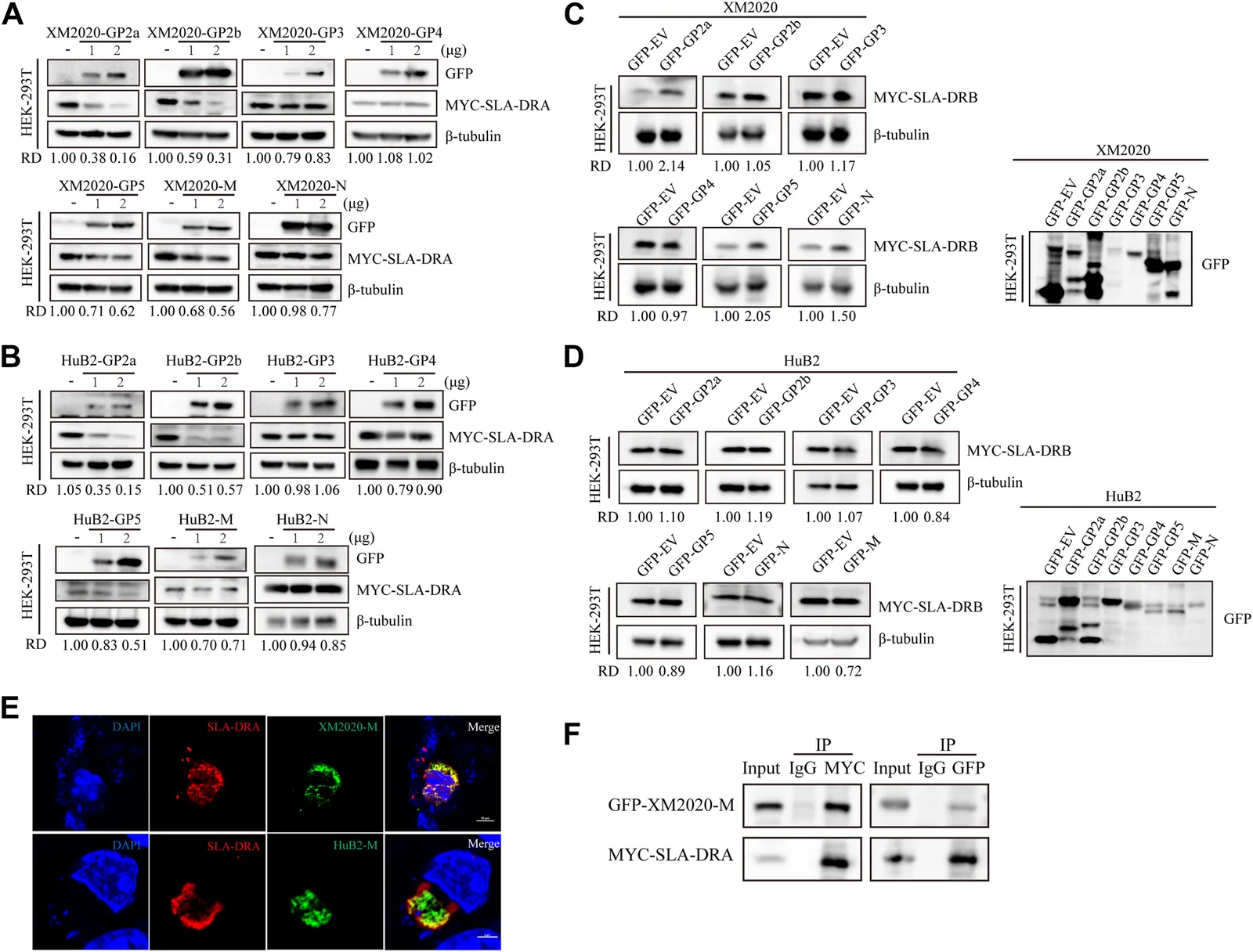
**XM2020-M downregulates SLA-DRA protein levels.***A*-*B*, HEK-293T cells were cotransfected with MYC-SLA-DRA and different doses of GFP- XM2020 (*A*) and HuB2 (*B*) structural proteins. Protein expression was analyzed by western blot with indicated antibodies. *C*-*D*, HEK-293T cells were cotransfected with MYC-SLA-DRB and different doses of GFP- XM2020 (*C*) and HuB2 (*D*) structural proteins. Protein expression was analyzed by western blot with the indicated antibodies. *E*, Marc-145 cells were cotransfected with MYC-SLA-DRA and GFP-XM2020-M or GFP-HuB2-M. At 24 h post transfection, IFA with the indicated antibodies and DAPI staining visualized fluorescence (the scale bar, represents 10 μm). *F*, HEK-293T cells were transfected with the indicated plasmids for 24 h. Co-IP and western blot analyses were performed with the indicated antibodies. Co-IP, Co-immunoprecipitation; DAPI, 4′,6-diamidino-2-phenylindole; IFA, immunofluorescence assay; SLA, swine leukocyte antigen.

### XM2020-M mediates SLA-DRA downregulation *via* the ubiquitin-proteasome degradation pathway

To determine the pathway responsible for SLA-DRA degradation, HEK-293T cells were cotransfected with SLA-DRA and XM2020-M. The cells were then treated with cycloheximide (CHX), a protein synthesis inhibitor (18). Western blot analysis showed that SLA-DRA levels remained stable following CHX treatment. In contrast, XM2020-M expression induced noticeable degradation (Fig. 4*A*), indicating that its degradation requires continuous XM2020-M expression.

**Figure 4:**
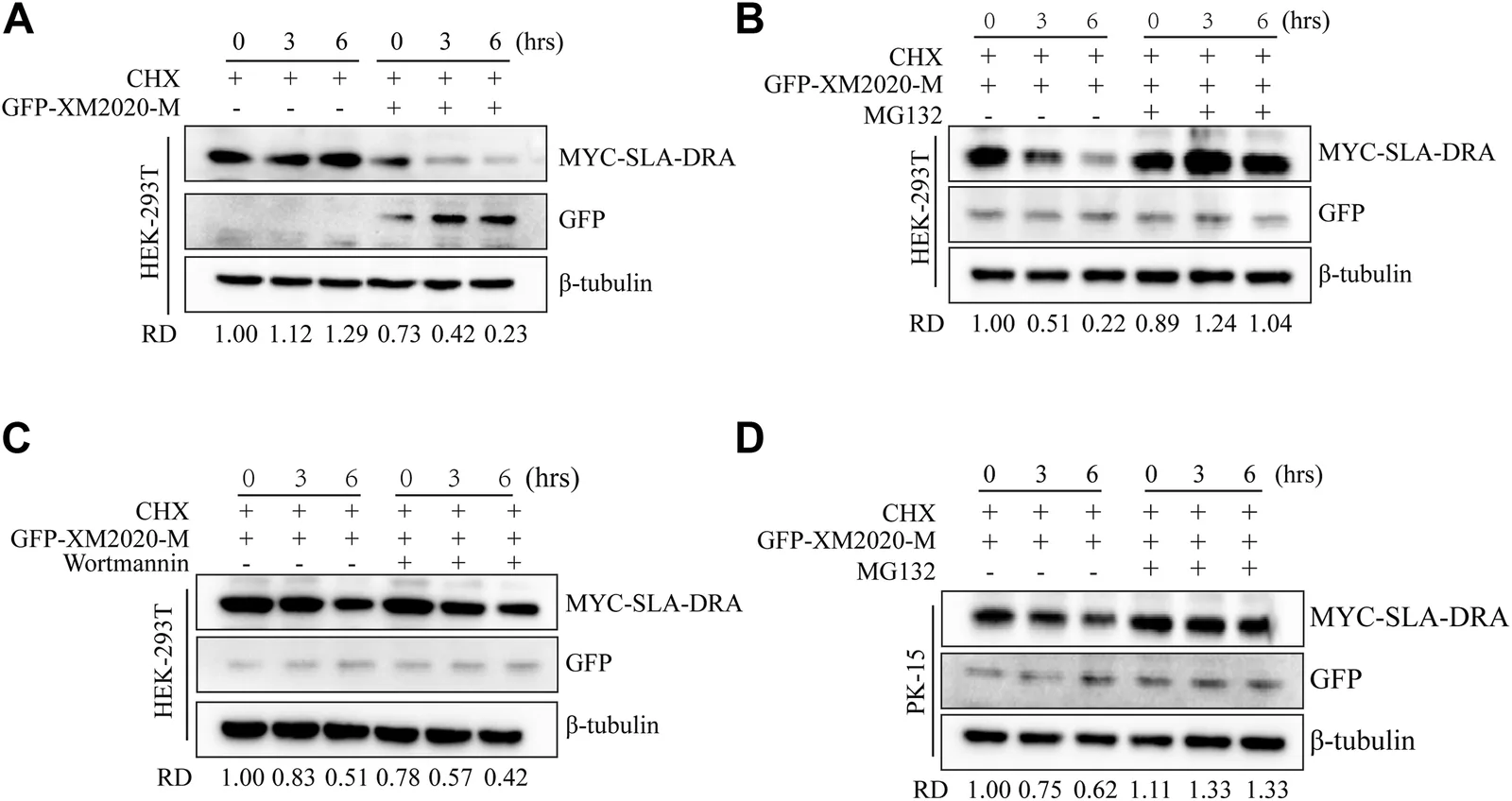
**XM2020-M downregulates SLA-DRA expression *via* the ubiquitin proteasome pathway.** (*A*-*C*) cycloheximide (CHX) chase assay reveals XM2020-M-mediated degradation of SLA-DRA. HEK-293T cells were transfected with MYC-SLA-DRA and GFP-XM2020-M, and then treated with CHX (50 μg/ml), MG132 (5 μM), wortmannin (10 μM), and DMSO. At 0, 3, and 6 h after treatment, cells were harvested for western blot to detect SLA-DRA protein levels. *D*, validation assay in PK-15 cells treated as in (*B*) with MG132 and CHX. DMSO, dimethyl sulfoxide; SLA, swine leukocyte antigen.

Since ubiquitination and autophagy are the major protein degradation pathways (25, 26), we next examined which pathway mediates SLA-DRA downregulation by XM2020-M. Treatment with the proteasome inhibitor MG132 effectively blocked XM2020-M-induced degradation of SLA-DRA. The autophagy inhibitor Wortmannin had no effect (Fig. 4, *B* and *C*). Similar results were observed in PK-15 cells (Fig. 4*D*), suggesting that this mechanism is conserved across cell types. Collectively, these results demonstrate that XM2020-M specifically downregulates SLA-DRA through the ubiquitin-proteasome degradation pathway.

### XM2020-M promotes K33-Linked polyubiquitination of SLA-DRA by recruiting MARCH6

To investigate the mechanism by which XM2020-M downregulates SLA-DRA, we first examined SLA-DRA ubiquitination. HEK-293T cells were cotransfected with MYC-SLA-DRA, GFP-XM2020-M, and a panel of hemagglutinin (HA)-tagged ubiquitin mutants. Immunoprecipitation (IP) followed by western blot showed that XM2020-M markedly increased SLA-DRA polyubiquitination. This effect was abolished by the K33R mutation, indicating K33-linkage specificity (Fig. 5*A*). K33-only ubiquitin confirmed that XM2020-M specifically promotes K33-linked polyubiquitination in a dose-dependent manner (Fig. 5*B*).

**Figure 5:**
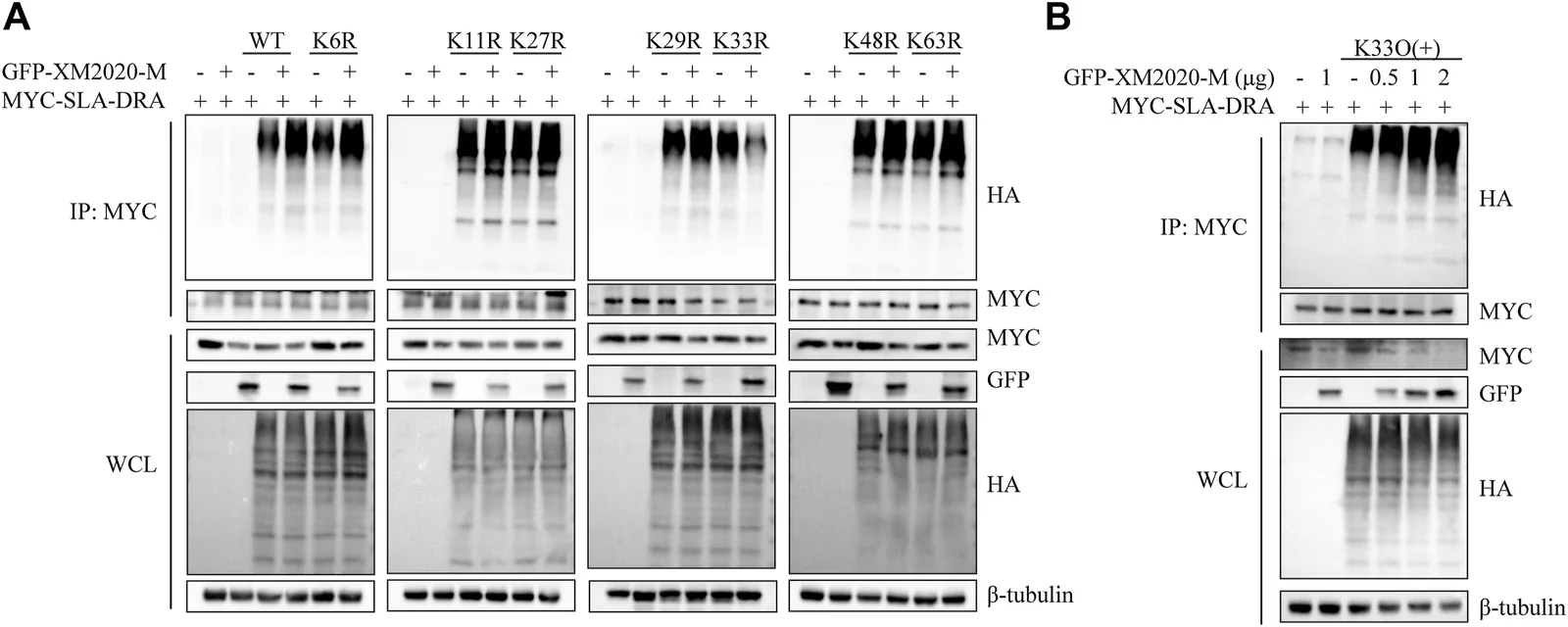
**XM2020-M enhances K33-linked polyubiquitination of SLA-DRA.***A*, the induction of site-specific ubiquitination of SLA-DRA was tested by cotransfecting HEK-293T cells with plasmids encoding GFP-XM2020-M, HA-tagged wild-type ubiquitin (WT-Ub) or mutant ubiquitin (K6R, K11R, K27R, K29R, K33R, K48R, and K63R), and either MYC-empty vector (EV) or MYC-SLA-DRA. After transfection, cell lysates were immunoprecipitated (Co-IP) and analyzed by western blot using the indicated antibodies to detect SLA-DRA ubiquitination. *B*, HEK-293T cells were cotransfected with MYC-SLA-DRA and HA-K33O, and treated with increasing concentrations of GFP-XM2020-M for 24 h. K33-linked ubiquitination levels were detected by immunoprecipitation and western blot to evaluate the dose-dependent effect. SLA, swine leukocyte antigen.

Next, we assessed the involvement of MARCH-family E3 ligases. The membrane-associated RING-CH (MARCH) family of E3 ubiquitin ligases regulates MHC molecules and thus is important in viral immune evasion (27, 28). Previously, MARCH8 and MARCH9 promoted human leukocyte antigen-DRA polyubiquitination (29). Transcriptomic analysis revealed that XM2020 treatment upregulated MARCH5 and MARCH6, while downregulating MARCH8 and MARCH9 (Fig. 6*A*). Given that XM2020-M specifically promoted K33-linked polyubiquitination of SLA-DRA, we next investigated whether any MARCH family member was involved in this process. Although XM2020-M overexpression increased mRNA levels of all four MARCH proteins, only MARCH6 protein abundance was elevated (Fig. 6, *B*–*D*). CHX chase assays further showed that XM2020-M stabilizes MARCH6 by preventing its degradation (Fig. 6*F*), and Co-IP and immunofluorescence analyses confirmed a direct interaction between XM2020-M and MARCH6 (Fig. 6, *H* and *I*). In contrast, HuB2-M had no effect on MARCH6 expression or stability (Fig. 6, *E* and *G*). These results identify MARCH6 as a key mediator of XM2020-M–induced downregulation of SLA-DRA.

**Figure 6:**
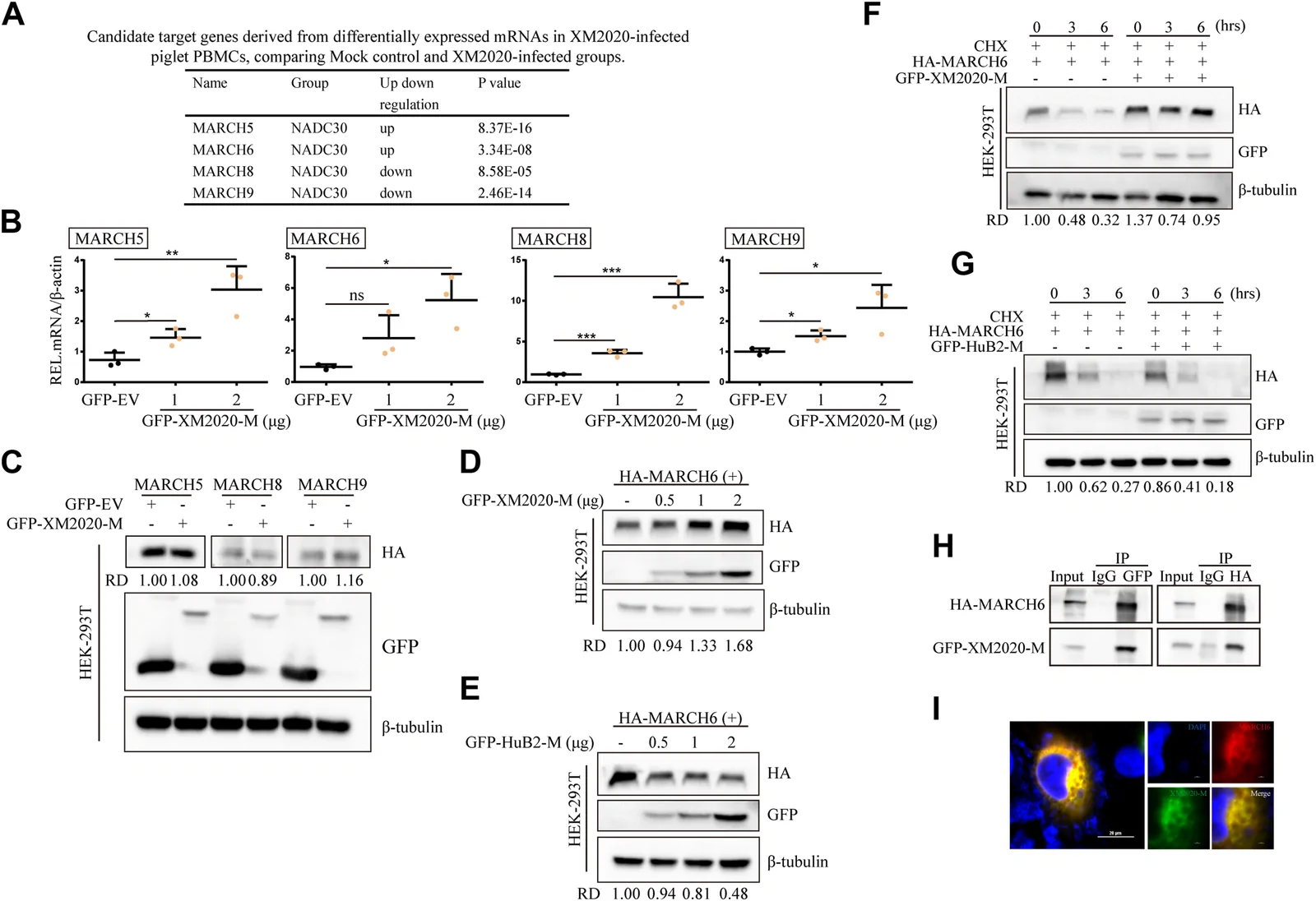
**XM2020-M interacts with MARCH6 and enhances its expression by inhibiting its protein degradation.***A*, transcriptomic analysis of MARCH family proteins. *B*-*C*, effect of XM2020-M on the expression of MARCH proteins. HEK-293T cells were transfected with different doses of GFP-XM2020-M for 24 h. Cell lysates were prepared for analysis of MARCH expression using qPCR (*B*) and western blot (*C*). Mean values ± SDs are shown (n = 3). Significance was assessed *via* two-tailed, unpaired Student’s t-tests. ns, not significant; ∗*p* < 0.05, ∗∗*p* < 0.01, ∗∗∗*p* < 0.001. *D*-*E*, HEK-293T cells were transfected with HA-MARCH6 and increasing amounts of GFP-XM2020-M (*D*) or GFP-HuB2-M (*E*) for 24 h, after which cell lysates were collected and analyzed for MARCH6 protein expression by western blot. *F*-*G*, HEK-293T cells were transfected with HA-MARCH6 and either GFP-XM2020-M (*F*), GFP-HuB2-M (*G*), or GFP-EV. Following transfection, cells were treated with CHX (50 μg/ml) and DMSO (control), then harvested at 0, 3, and 6 h for western blot analysis of MARCH6 protein levels. *H*, HEK-293T cells were transfected with the indicated plasmids for 24 h, then subjected to Co-IP and western blot analyses with the relevant antibodies. *I*, Marc-145 cells were cotransfected with GFP-XM2020-M and HA-MARCH6. At 24 h post transfection, cells were processed for IFA analysis with anti-HA antibodies and visualized after DAPI nuclei staining. The scale bars represent 20 μm. CHX, cycloheximide; qPCR, quantitative PCR; DAPI, 4′,6-diamidino-2-phenylindole; DMSO, dimethyl sulfoxide; IFA, immunofluorescence assay.

To confirm the role of MARCH6, Co-IP assays showed that MARCH6 specifically interacts with SLA-DRA (Fig. 7*A*). Co-expression of XM2020-M with MARCH6 and SLA-DRA reduced SLA-DRA levels in a dose-dependent manner. MARCH6 alone had no effect (Fig. 7, *B* and *C*). Knockdown of MARCH6 partially restored SLA-DRA levels in the presence of XM2020-M or after XM2020 infection (Fig. 7, *D* and *E*). HuB2-M did not affect SLA-DRA stability (Fig. 7*B*). Collectively, these findings demonstrate that XM2020-M enhances MARCH6 E3 ligase activity, promoting SLA-DRA degradation *via* K33-linked polyubiquitination. Thus, XM2020-M requires MARCH6 to mediate the targeted ubiquitination and degradation of SLA-DRA.

**Figure 7:**
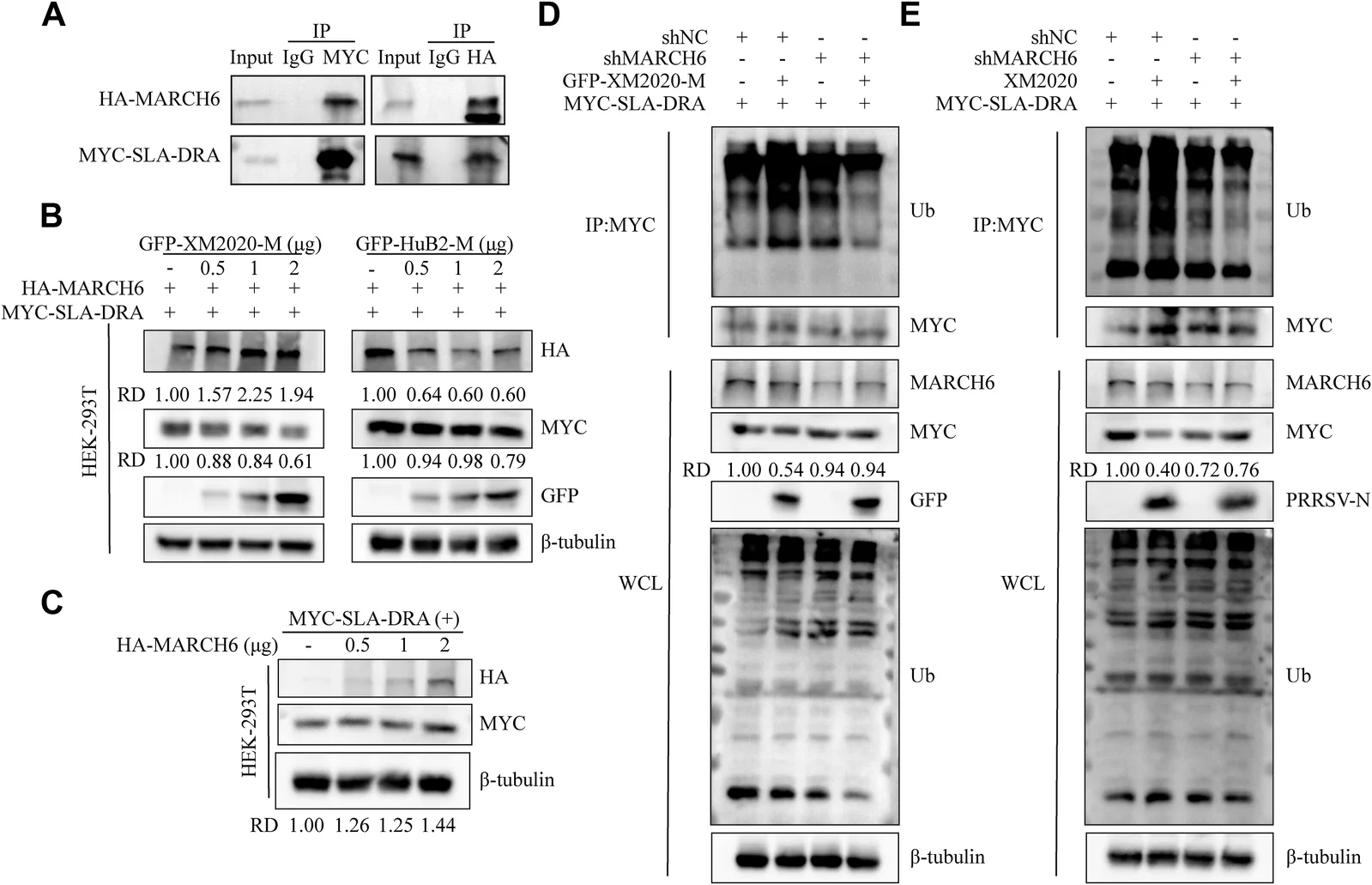
**XM2020-M-mediated SLA-DRA downregulation through the MARCH6 pathway.***A*, HEK-293T cells were cotransfected with MARCH6 and MYC-SLA-DRA. At 24 h post transfection, Co-IP and western blot analyses were performed with the indicated antibodies. *B*, HEK-293T cells were transfected with different doses of GFP-XM2020-M or GFP-HuB2, together with MYC-SLA-DRA and HA-MARCH6. At 24 h post transfection, the cells were harvested for western blot to detect SLA-DRA and MARCH6 protein levels. *C*, HEK-293T cells were transfected with MYC-SLA-DRA and different doses of HA-MARCH6. At 24 h post transfection, the cells were harvested for western blot to detect SLA-DRA protein levels. *D*, SLA-DRA degradation by XM2020-M in shMARCH6 *versus* shCT cells. HEK-293T cells were transfected with the NC or shMARCH6-2, MYC-SLA-DRA, and GFP-XM2020-M. At 24 h post transfection, the cells were subjected to IP and western blot with the indicated antibodies. *E*, SLA-DRA degradation by XM2020 infection in shMARCH6 *versus* shCT cells. Marc-145 cells were transfected with the NC or shMARCH6-2 and MYC-SLA-DRA for 24 h, then infected with XM2020 (MOI=1). Cell lysates were then subjected to IP and western blot with the indicated antibodies. Co-IP, co-immunoprecipitation; SLA, swine leukocyte antigen.

### XM2020-M 93rd amino acid residue is critical for SLA-DRA degradation

To identify the key domains within XM2020-M responsible for SLA-DRA degradation, we constructed a series of truncation mutants (Fig. 8*A*). Western blot analysis showed that the GFP-XM2020-M-139aa, -118aa, and -98aa mutants increased intracellular endogenous ubiquitin levels, whereas the GFP-XM2020-M-78aa mutant lacked this capacity (Fig. 8*A*). CHX chase assays further confirmed that the 78–98aa region is essential for XM2020-M to stabilize MARCH6 (Fig. 8*B*). This highlights the functional importance of the 78–98aa region of XM2020-M in regulating SLA-DRA degradation.

**Figure 8:**
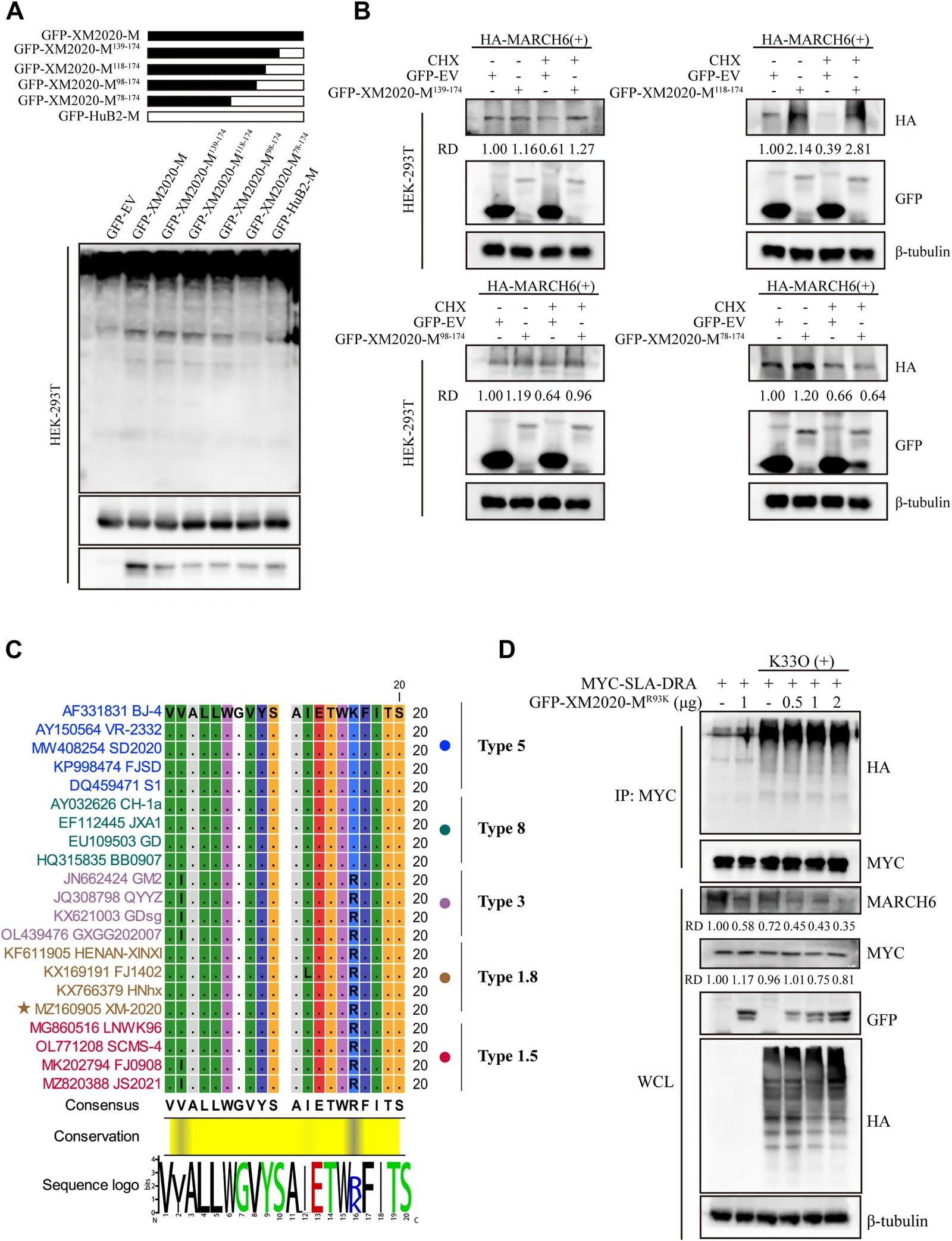
**XM2020-M 93rd amino acid residue is critical for SLA-DRA degradation.***A*, identify functional domain differences between XM2020-M and HuB2-M. The top diagram shows the construction of XM2020-M protein mutants. HEK-293T cells were transfected with HA-MARCH6 and GFP-XM2020-M mutants. Cell lysates collected after 24 h were analyzed by western blot with the indicated antibodies (*bottom*). *B*, HEK-293T cells were transfected with HA-MARCH6 and GFP-XM2020-M mutants. Cells were treated with CHX (50 μg/ml) for 6 h, then harvested for western blot to detect MARCH6 protein levels. *C*, analyze conservation of M protein residues 78 to 98 across PRRSV lineages. *D*, HEK-293T cells were cotransfected with MYC-SLA-DRA, HA-K33O, and increasing doses of the GFP-XM2020-M R93K mutant. At 24 h post transfection, cell lysates were collected for immunoprecipitation and western blot analysis with the indicated antibodies. The R93K mutation impaired XM2020-M-mediated degradation of SLA-DRA and reduced K33-linked ubiquitination levels. CHX, cycloheximide; PRRSV, porcine reproductive and respiratory syndrome virus; SLA, swine leukocyte antigen.

Sequence alignment revealed a residue divergence at position 93 of XM2020-M (arginine, R, in XM2020-M *versus* lysine, K, in HuB2) (Fig. 8*C*). This position is conserved among PRRSV lineages 3, 1.8, and 1.5, but shows marked divergence from lineages 5 and 8. Although our functional analysis was performed using the XM2020 strain, variation at this position may help explain lineage-specific differences in immunomodulatory functions.

To verify its functional significance, we cotransfected HEK-293T cells with increasing amounts of the XM2020-M-R93K mutant along with HA-K33O and MYC-SLA-DRA. As shown in Figure 8*D*, the R93K mutation reduced SLA-DRA degradation, accompanied by a decrease in endogenous MARCH6 expression levels. Immunoprecipitation results indicated that K33-linked ubiquitination levels were not increased. These findings suggest that the 93rd amino acid residue is critical for XM2020-M-mediated regulation of MARCH6 and SLA-DRA ubiquitination. Therefore, residue 93 is a key determinant of XM2020-M's regulatory function in this pathway.

## Discussion

Since first emerging in China in 2013, NADC30-like PRRSV has become the main circulating strain and a focus of research. These strains show reduced pathogenicity compared to HP-PRRSV variants like HuB2. However, we found they have advanced immune evasion mechanisms that likely aid their spread. This study demonstrates that the NADC30-like PRRSV strain XM2020 downregulates MHC class II molecules on PBMCs through multiple structural proteins, thereby suppressing host immunity. We acknowledge that PBMCs are heterogeneous and APCs represent a minor fraction, which may dilute APC-derived signals. PBMCs also lack tissue-resident APCs. Despite these limitations, they offer practical advantages for longitudinal monitoring, including convenient blood sampling, repeated sampling from the same animal, compliance with the 3R principle, and reduced animal use. In addition, PBMCs have been used as a cellular model in previous PRRSV studies (30, 31, 32). Using this model, we found that both infectious and UV-inactivated XM2020 particles induced equivalent SLA-DR suppression in PBMCs. This indicates that the immune evasion mechanism operates independently of viral replication. This replication-independent feature distinguishes XM2020 from previously characterized PRRSV strains, which rely on nonstructural proteins for MHC downregulation (33, 34, 35, 36). Notably, the *in vitro* downregulation of SLA-DRB by XM2020 was not observed *in vivo*, suggesting that microenvironmental cues or regulatory pathways distinct from those governing SLA-DRA may be involved. This intriguing disparity calls for further investigation. Collectively, these findings reveal a distinct immunosuppressive strategy employed by NADC30-like PRRSV and provide a plausible explanation for the clinical association between such infections and increased susceptibility to secondary pathogens.

Central to this immune evasion strategy is the novel interaction between XM2020-M, the host E3 ubiquitin ligase MARCH6, and SLA-DRA. Ubiquitination serves as a fundamental mechanism for intracellular protein degradation (37), regulating diverse cellular processes including cell cycle progression, apoptosis, and signal transduction. MARCH-family E3 ubiquitin ligases are known to mediate MHC class II regulation (38). Our data demonstrate that XM2020-M specifically promotes K33-linked polyubiquitination of SLA-DRA through recruitment and stabilization of MARCH6, marking the MHC II complex for proteasomal degradation. This represents a significant advance in our understanding of viral immune evasion, being the first demonstration of viral exploitation of MARCH6 for immune modulation. Prior to this work, MARCH6’s characterized functions were limited to cancer progression through ubiquitin-mediated protein regulation (39, 40). And MARCH6 as a key metabolic regulator of lipid metabolism, including cholesterol biosynthesis and lipid droplet homeostasis (41, 42, 43), with no established connection to immune regulation. The discovery that XM2020-M stabilizes MARCH6 by inhibiting its autodegradation reveals a new paradigm in host–pathogen interaction, where viral proteins can selectively manipulate the abundance of host E3 ligases to achieve immune suppression (Fig. 9).

**Figure 9:**
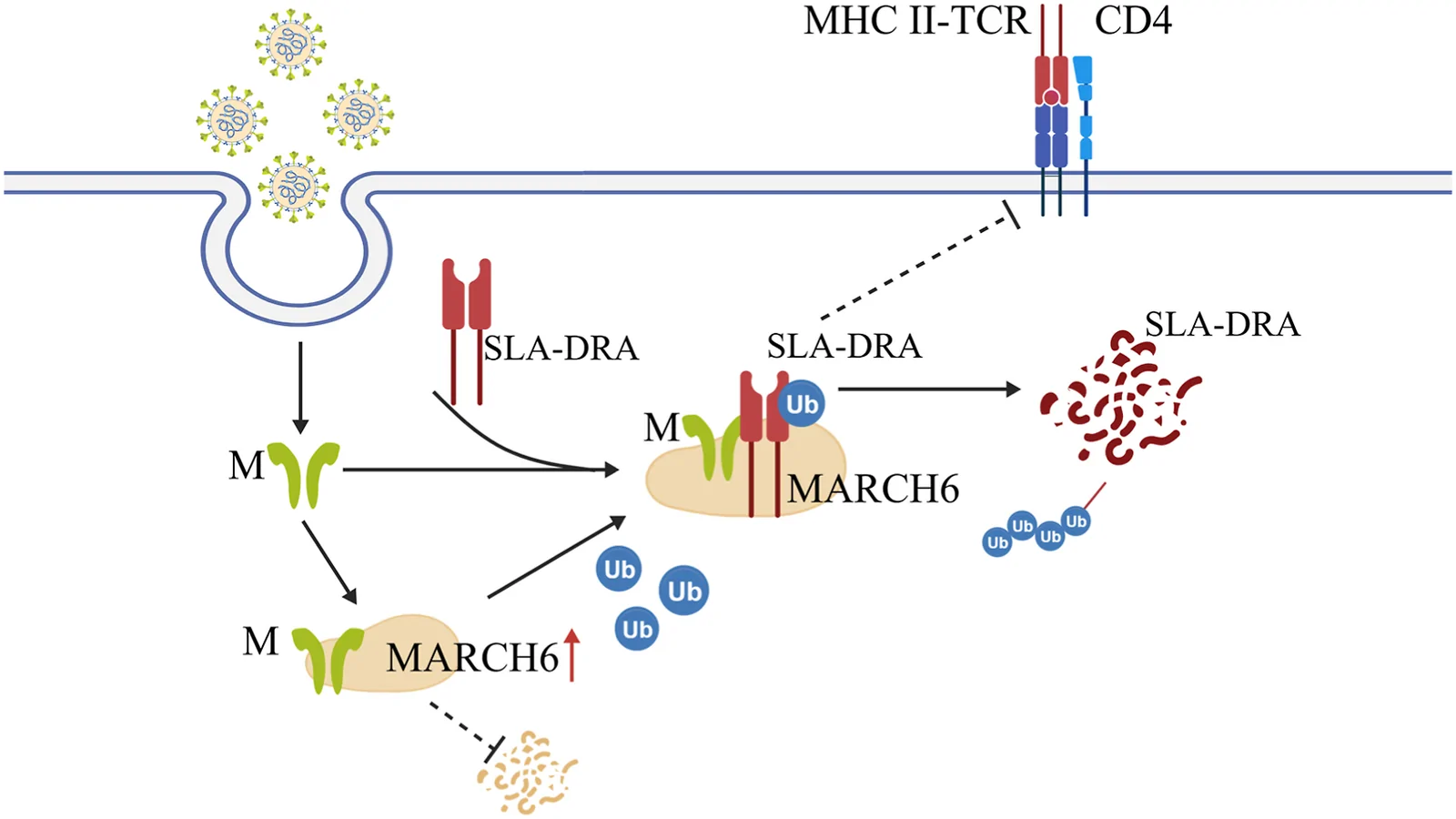
**A proposed pathway for XM2020-M-mediated K33-linked polyubiquitination of SLA-DRA.** XM2020-M binds SLA-DRA and recruits the E3 ubiquitin ligase MARCH6 to promote SLA-DRA degradation *via* the ubiquitin-proteasome pathway. XM2020-M also stabilizes MARCH6 by blocking its autodegradation, further enhancing SLA-DRA downregulation. SLA, swine leukocyte antigen.

The evolutionary implications of these findings are noteworthy. The acquisition of M protein domains enabling MARCH6 interaction appears to be a recent adaptation in NADC30-like strains, providing them with a selective advantage over earlier PRRSV variants. This sophisticated mechanism contrasts with the more direct immune suppression mediated by HP-PRRSV strains, potentially explaining the distinct immunological profiles observed between these PRRSV lineages. The broader significance of MHC class II modulation in viral pathogenesis is underscored by parallel observations in other systems, including SARS-CoV-2 (44, 45, 46, 47), suggesting this may represent a conserved strategy among diverse viruses.

The ability of NADC30-like PRRSV to suppress MHC class II expression is crucial. It allows the virus to dampen adaptive immune responses. Immunosuppressive domains remain in structural proteins, even in inactivated vaccine preparations. This may limit the effectiveness of current vaccines against NADC30-like strains. Our work provides a molecular plan for next-generation vaccines. Targeted modification of the M protein functional domain could overcome this barrier in PRRSV vaccine design. Furthermore, the identification of MARCH6 as a central player in this immune evasion pathway opens new avenues for therapeutic intervention, not only for PRRSV but potentially for other viral infections where MHC class II modulation contributes to pathogenesis.

## Experimental procedures

### Ethics approval and consent to participate

All animals were strictly handled in accordance with the Good Animal Practice of the People’s Republic of China Animal Ethics Procedures and Guidelines. All animal experiments were approved by the Ethics of Animal Experiments of Northwest A&F University (approval number DY2025025).

### Cell, viruses, plasmid, antibodies, and reagents

The MARC-145, HEK-293T, and PK-15 cells, which are types of mammalian cell lines commonly used in laboratory research, were maintained in Dulbecco’s modified Eagle’s medium (DMEM; Biological Industries) with 10% fetal bovine serum (FBS; Biological Industries) under standard conditions. PAMs, which are immune cells obtained from the lungs of pigs, were prepared from lung lavage samples of 4-week-old healthy piglets. These cells were grown in RPMI 1640 medium containing 10% fetal bovine serum and kept at 37 °C with 5% CO_2_. All cells were negative for *mycoplasma*. PBMCs, a group of blood cells with round nuclei, such as lymphocytes and monocytes, were isolated from whole blood samples by density gradient centrifugation using Ficoll-Paque (P4420; Solarbio) according to the manufacturer's instructions.

This research study included two PRRSV isolates: the NADC30-like strain XM2020 (GenBank accession number PQ623172.1) and the HP-PRRSV strain HuB2 (GenBank accession number EF112446.1). The median tissue culture infectious dose (TCID50, a measure of the amount of virus required to infect 50% of cultured cells) was determined using MARC-145 cells (a monkey kidney cell line commonly used for PRRSV studies).

For molecular studies, we cloned all structural protein genes from both PRRSV XM2020 and HuB2 into the pEGFP-C1 or -N1 expression vector. We amplified complementary DNA (cDNA) sequences coding for MHC class II molecules (proteins important for immune responses) and MARCHF (a family of E3 ubiquitin ligases) from total PAMs cDNA, then inserted them into pCMV-MYC and pCDNA3.1-HA vectors. Table 1 outlines all primer sequences and respective cloning methods. We introduced plasmids (circular DNA molecules used for gene expression) into HEK-293T, PK-15, and Marc-145 cells using TurboFect Transfection Reagent (Thermo Fisher Scientific) according to the manufacturer's instructions.

**Table 1:** List of primers for cloning

Primer	Sequences(5′-3′)	Description
SLA-DRA-F	TGCTTATGGCCATGGAGGCCATGACCATACTTGGGGTCCC	Cloning of SLA-DRA
SLA-DRA-R	GATCCCCGCGGCCGCGGTACTCACAGAGGCCCTCGGCGTT	Cloning of SLA-DRA
SLA-DRB-F	TTATGGCCATGGAGGCCCGAATGTTGCATCTGTGTTTCTC	Cloning of SLA-DRB
SLA-DRB-R	CGCGGCCGCGGTACCTCGAGTCAGCTCAGGAGGCCTGTTG	Cloning of SLA-DRB
MARCH5-F	CAGATTACGCTGGATCCCCGATGCCGGACCAAGCCCTACA	Cloning of MARCH5
MARCH5-R	CCCTCTAGATGCATGCTCGACTATGCTTCTTCTTGCTCTG	Cloning of MARCH5
MARCH6-F	CAGATTACGCTGGATCCCCGATGGACACCGCCGAGGAAGA	Cloning of MARCH6
MARCH6-R	CCCTCTAGATGCATGCTCGATTATTCTTGGGATGACTGTGGAGG	Cloning of MARCH6
XM2020-GP2a-F	CCGGACTCAGATCTCGAGCTATGAAATGGGGGCCTTGCAG	Cloning of XM2020-GP2a
XM2020-GP2a-R	CCGCGGTACCGTCGACTGCATCACCATGAGCTCGAAGGAA	Cloning of XM2020-GP2a
XM2020-GP2b-F	TACAAGTCCGGACTCAGATCAATGGGGGCCTTGCAGAGCAT	Cloning of XM2020-GP2b
XM2020-GP2b-R	ACCGTCGACTGCAGAATTCGTCATAGGATCTTCTGTAATTGCTCAG	Cloning of XM2020-GP2b
XM2020-GP3-F	AGCGCTACCGGACTCAGATCTCGAGATGGTTAATAGTTGTGCACT	Cloning of XM2020-GP3
XM2020-GP3-R	CGACTGCAGAATTCGAAGCTTTCGCCGCGCGACATTGAGGG	Cloning of XM2020-GP3
XM2020-GP4-F	AGCGCTACCGGACTCAGATCTCGAGATGGCTGCGTCCTTTCTTTT	Cloning of XM2020-GP4
XM2020-GP4-R	CGACTGCAGAATTCGAAGCTTAATTGCCAACAGAATGGCAA	Cloning of XM2020-GP4
XM2020-GP5-F	GGACTCAGATCTCGAGCTCAAGCTATGTTGGGGAAATGCTTGAC	Cloning of XM2020-GP5
XM2020-GP5-R	GTACCGTCGACTGCAGAATTCCTATGGACGACCCCATTGTT	Cloning of XM2020-GP5
XM2020-M-F	CGGACTCAGATCTCGAGCTCAATGGGGTCGTCCATAGACGA	Cloning of XM2020-M
XM2020-M-R	GTCGACTGCAGAATTCGAAGTTTGGCATATTTAACAAGGT	Cloning of XM2020-M
XM2020-N-F	AGCTGTACAAGTCCGGACTCATGCCAAATAACAACGGCAG	Cloning of XM2020-N
XM2020-N-R	ACCGTCGACTGCAGAATTCGTCATGCTGAGGGTGACGTTG	Cloning of XM2020-N
HuB2-GP2a-F	ACAAGTCCGGACTCAGATCTATGAAATGGGGTCTATGCAA	Cloning of HuB2-GP2a
HuB2-GP2a-R	CCGCGGTACCGTCGACTGCATCACCATGAGTTCAAAAGAA	Cloning of HuB2-GP2a
HuB2-GP2b-F	TTTAGTGAACCGTCAGATCCATGGGGTCTATGCAAAGCCT	Cloning of HuB2-GP2b
HuB2-GP2b-R	TGCAGAATTCGAAGCTTGAGTCATAAGATCTTCTGTAATT	Cloning of HuB2-GP2b
HuB2-GP3-F	CCGGACTCAGATCTCGAGCTATGGCTGATAGCTGTACATT	Cloning of HuB2-GP3
HuB2-GP3-R	CCGCGGTACCGTCGACTGCACTATCGCCGTGCGGCACTGA	Cloning of HuB2-GP3
HuB2-GP4-F	CCGGACTCAGATCTCGAGCTATGGCTGCGCCCTTTCTTTT	Cloning of HuB2-GP4
HuB2-GP4-R	CCGCGGTACCGTCGACTGCATCAAATTGCCAGTAGGATGG	Cloning of HuB2-GP4
HuB2-GP5-F	GGACTCAGATCTCGAGCTCAAATGTTGGGGAAGTGCTTGAC	Cloning of HuB2-GP5
HuB2-GP5-R	TTATCTAGATCCGGTGGATCCCTAGAGACGACCCCATTGTT	Cloning of HuB2-GP5
HuB2-M-F	GGACTCAGATCTCGAGCTCAAATGGGGTCGTCTCTAGACGA	Cloning of HuB2-M
HuB2-M-R	TTATCTAGATCCGGTGGATCCTTATTTGGCATATTTAACAA	Cloning of HuB2-M
HuB2-N-F	AGCGCTACCGGACTCAGATCATGCCAAATAACAACGGCAA	Cloning of HuB2-N
HuB2-N-R	CGACTGCAGAATTCGAAGCTTTGCTGAGGGTGATGCTGTGG	Cloning of HuB2-N
XM2020-HuB2-78-174-F	TTGCACTTACCATGGGGGCTGTAGTTGCACTTCTTTGGGG	Cloning of XM2020-M/HuB2-M chimeric mutants
XM2020-HuB2-78-174-R	CCCCAAAGAAGTGCAACTACAGCCCCCATGGTAAGTGCAA	Cloning of XM2020-M/HuB2-M chimeric mutants
XM2020-HuB2-98-174-F	CCTGGAGATTCATCACCTCCAGATGCCGTTTGTGCTTGCT	Cloning of XM2020-M/HuB2-M chimeric mutants
XM2020-HuB2-98-174-R	AGCAAGCACAAACGGCATCTGGAGGTGATGAATCTCCAGG	Cloning of XM2020-M/HuB2-M chimeric mutants
XM2020-HuB2-118-174-F	CCCCTGCCCACCACGTTGAAAGTGCCGCGGGCTTTCATCC	Cloning of XM2020-M/HuB2-M chimeric mutants
XM2020-HuB2-118-174-R	GGATGAAAGCCCGCGGCACTTTCAACGTGGTGGGCAGGGG	Cloning of XM2020-M/HuB2-M chimeric mutants
XM2020-93aa-F	TCAGCCATTGAAACCTGGAAATTCATCACCTCCAGATGCC	Cloning of XM2020-93 R/KHuB2 chimeric mutants
XM2020-93aa-R	GGCATCTGGAGGTGATGAATTTCCAGGTTTCAATGGCTGA	Cloning of XM2020-M-93 R/KHuB2 chimeric mutants

In this study, the following antibodies were used. A mouse anti-PRRSV-N polyclonal antibody was produced in our laboratory. Horseradish peroxidase (HRP)-conjugated Goat Anti-Mouse IgG (H + L) (Proteintech, SA00001-1), HRP-conjugated Goat Anti-Rabbit IgG (H + L) (Proteintech, SA00001-2), rabbit anti-MYC polyclonal antibody (Proteintech, 16286-1-AP), and mouse anti-β-tubulin monoclonal antibody (Proteintech, 66240-1-Ig) were purchased commercially. Other antibodies included mouse anti-MYC monoclonal antibody (Yeasen, 30601ES20), rabbit anti-MARCH6 polyclonal antibody (Affinity Biosciences, DF9482) and HA-Tag (C29f4) Rabbit mAb (Cell Signaling Technology, 3724). For detecting proteins by immunofluorescence, we used goat anti-rabbit IgG (YSFluor 594 AffiniPure). The mouse anti-SLA-DR monoclonal antibody was kindly provided by Professor Yuchen Nan (Northwest A&F University).

MG132 (a proteasome inhibitor, T2154), CHX (cycloheximide, a protein synthesis inhibitor, T1225), and Wortmannin (a PI3K inhibitor, T6283) were purchased from TargetMOI.

### Animals and inoculations

Fifteen 6-week-old Landrace piglets from a local farm in Xianyang, Shanxi, China, were confirmed free of PRRSV, porcine circovirus type 2, pseudorabies virus, and porcine epidemic diarrhoea virus (PEDV) before the study. The piglets were divided into three groups: control (no treatment, n = 5), HuB2 (n = 5), and XM-2020 (n = 5). To prevent cross-contamination, all piglets were housed in separate biosafety-level isolation rooms.

Animals in the infection groups received 3 ml of either the HuB2 or XM-2020 strain *via* intramuscular injection, each at 5 × 10^5^ plaque-forming units (PFUs). Blood samples were collected on days 3, 5, 7, and 14 after inoculation for virological and immunological tests. The VDPro PRRSV AB ELISA Kit was used to detect antibodies against the PRRSV nucleocapsid (N) protein.

All procedures followed institutional animal care and use guidelines. At 42 days post infection, all animals were humanely euthanized.

### Western blot analysis

PBMCs, human embryonic kidney 293T (HEK-293T) cells, and MARC-145 (monkey kidney) cells were lysed to extract total proteins using radioimmunoprecipitation assay buffer supplemented with 1% phenylmethylsulfonyl fluoride (PMSF, a protease inhibitor) and phosphatase inhibitors. After lysis, samples were mixed with 5× sodium dodecyl sulfate-polyacrylamide gel electrophoresis (SDS-PAGE) loading buffer and heated for 10 min to denature proteins. Proteins were then separated on a 10% SDS-PAGE gel and transferred onto nitrocellulose membranes. The membranes were blocked for 1 h at room temperature with 10% nonfat milk in Tris-buffered saline with 0.1% Tween 20 (TBST) to prevent nonspecific binding. After blocking, primary antibodies were incubated with the membranes overnight at 4 °C. After washing to remove unbound antibodies, the membranes were incubated for 1 h at room temperature with HRP-conjugated secondary antibodies. Protein detection was performed using an enhanced chemiluminescence (ECL) imaging device, which allows visualization of HRP-catalyzed light emission.

HEK-293T cells, a human embryonic kidney cell line, were treated with 50 μg/ml cycloheximide (Sigma-Aldrich), an inhibitor of protein synthesis. Cells were collected at predetermined intervals after treatment. The lysates were then subjected to western blotting, a technique used to detect specific proteins, to evaluate the kinetics of SLA-DRA breakdown.

### RNA isolation, transcriptomic analysis, and qPCR

Total RNA was extracted from PBMCs or other samples using Thermo Fisher Scientific's TRIzol reagent according to the manufacturer's instructions. To ensure experimental repeatability, three biological replicates were included in PBMC analysis, with or without PRRSV infection, using BMKCloud for transcriptome profiling. Subsequently, cDNA synthesis was performed using the Cofitt One Plus cDNA First Strand Synthesis Kit (Cofitt) according to the manufacturer's instructions, and qPCR was carried out with the 2× Fast qPCR Master Mixture (Green) (DiNing). The housekeeping gene β-actin was amplified as an internal control to normalize RNA input levels. All primer sequences used for qPCR amplification are listed in Table 2. Relative gene expression levels were determined using the comparative threshold cycle (2−ΔΔCT) approach.

**Table 2:** List of primers for qPCR

Primer	Sequences(5′-3′)	Description
SLA-DRα-F	GCCCTGAAGCCACTCTAA	For qPCR detection
SLA-DRα-R	GGAAAGCCAGCACAAGAA	For qPCR detection
SLA-DRβ-F	GAGGGCACGGTCTGAATC	For qPCR detection
SLA-DRβ-R	AGGGCGTCCTTTCTGATT	For qPCR detection
GAPDH-F	TCGCCATCAATGACCCCTTC	For qPCR detection
GAPDH-R	CACCCCATTTGATGTTGGCG	For qPCR detection
PRRSV-N-F	AAACCAGTCCAGAGGCAAGG	For qPCR detection
PRRSV-N-R	GCAAACTAAACTCCACAGTGTAA	For qPCR detection
MARCH5 (HEK-293T)-F	CTGGACAGAAGTTGCTGGGT	For qPCR detection
MARCH5 (HEK-293T)-R	GCCACTCTGGCTGTACTGTT	For qPCR detection
MARCH6 (HEK-293T)-F	ATGGACACCGCGGAGGAA	For qPCR detection
MARCH6 (HEK-293T)-R	GTGCACGTCACCACAAAACA	For qPCR detection
MARCH8 (HEK-293T)-F	CTGCTGTGAGCTGTGCAAGT	For qPCR detection
MARCH8 (HEK-293T)-R	GATGGCGATGACATGGAACG	For qPCR detection
MARCH9 (HEK-293T)-F	TCATCAGGTCAAGGATAGTC	For qPCR detection
MARCH9 (HEK-293T)-R	CCACACTGTATTTGGTGTCTAGG	For qPCR detection
HEK-GAPDH-F	GTCGTCGACAACGGCTCCG	For qPCR detection
HEK-GAPDH-R	ATTGTAGAAGGTGTGGTGC	For qPCR detection
Marc-GAPDH-F	CAAGAAGGTGGTGAAGCA	For qPCR detection
Marc-GAPDH-R	AAGGTGGAAGAGTGGGTG	For qPCR detection
IRF4-D-F	ACGCTGTGCTCTGAACAAGA	For qPCR detection
IRF4-D-R	GAGTGAGGTGTAAGGGGCTG	For qPCR detection
SLC2A3-D-F	GCTCTTCAGAGCACCCAACT	For qPCR detection
SLC2A3-D-R	CTCCTGAACACCGGCATCTT	For qPCR detection
BHLHE40-D-F	TCGGGACCTGAAATCTTCGC	For qPCR detection
BHLHE40-D-R	GTTTTTGCCTGGGCCTTCAG	For qPCR detection
DDIT4-D-F	TTCGTCCTTGTCCCGAACTC	For qPCR detection
DDIT4-D-R	CCCCATCCAGGTATGCAGAG	For qPCR detection
HSP70.2-D-F	ATGGCGAAGAGCGTGGC	For qPCR detection
HSP70.2-D-R	ATGGCGAAGAGCGTGGC	For qPCR detection
IL2RA-D-F	AGAAACTCAGGGCCAGATGC	For qPCR detection
IL2RA-D-R	TTGTTCTGGGCAGACCCATC	For qPCR detection
BCL2L1-D-F	GGGACTGAATCAGAAGCGGA	For qPCR detection
BCL2L1-D-R	CAGTTCAAACTCATCGCCCG	For qPCR detection
ALDOC-D-F	GCAACAGGACAACGCTGG	For qPCR detection
ALDOC-D-R	AGGCGTGGTTAGCAATGTAGA	For qPCR detection
MMP9-D-F	TGACCGACAGAAAGCAGTCC	For qPCR detection
MMP9-D-R	CGCCCTCAAAGGTCTGGAAT	For qPCR detection
GPNMB-D-F	TTGTCTCTATCCCCGGCAGA	For qPCR detection
GPNMB-D-R	TGACCACGATCCCAGTGTTG	For qPCR detection
ANXA1-D-F	AGCCTCCCATAAAGCGATAACA	For qPCR detection
ANXA1-D-R	CTTCATCCAGGGGCTTTCCT	For qPCR detection
CCL2-D-F	ATCTTCAAGACCATCGCGGG	For qPCR detection
CCL2-D-R	TCAAGGCTTCGGAGTTTGGTT	For qPCR detection
HMOX1-D-F	GACTCCACTCCCGTGACAAC	For qPCR detection
HMOX1-D-R	TCACATGGCATAAAGCCCCA	For qPCR detection
SDC2-D-F	AGGGCGTAGAGAGTCCAGAG	For qPCR detection
SDC2-D-R	TTCTGCATCTTGTCCAGCGT	For qPCR detection
HEXA-D-F	ACCTCACCAAAAAGGGCTCC	For qPCR detection
HEXA-D-R	TCTGCCAACACACGGATACC	For qPCR detection
FCGRT-D-F	CTGGTCGTCGCTAACAGTCA	For qPCR detection
FCGRT-D-R	GGAAGCCGACCATGATTCCA	For qPCR detection

### IP and protein ubiquitination assay

HEK-293T cells were transfected with the indicated plasmids and harvested after 24 h. Cells were lysed in ice-cold IP lysis buffer containing protease inhibitors and 1 mM PMSF for 15 min at 4 °C. Lysates were centrifuged at 12,000*g* for 15 min, and supernatants were collected. These were incubated with primary antibodies overnight at 4 °C with gentle rotation. Protein A/G magnetic beads were then added for a 2-h incubation at 4 °C. Bead-bound complexes were washed five times with PBS containing 0.1% Tween 20 (PBST), resuspended in 2× SDS-PAGE loading buffer, and denatured by boiling at 95 °C for 10 min before western blot. SLA-DRA ubiquitination was then assessed using a modified immunoprecipitation-based ubiquitination assay, preparing all samples to preserve ubiquitin modifications (34).

### RNAi

Chemically synthesized shRNAs for RNA interference (RNAi) assays were obtained from Sangon Biotech. Endogenous MARCH6 was knocked down by transfecting the indicated MARCH6 shRNA using TurboFect Transfection Reagent (Thermo Fisher Scientific).

The shRNA sequences targeted for shMARCH6 are as follows:

MARCHF6-2-F CACCTTAGTCCATCGGCGGATTTATTTCAAGAGAATAAATCCGCCGATGGACTAATTTTTTG.

MARCHF6-2-R GATCCAAAAAATTAGTCCATCGGCGGATTTATTCTCTTGAAATAAATCCGCCGATGGACTAA.

### Immunofluorescence assay

MARC-145 cells were transfected with experimental plasmids for 24 h. Cells were fixed with 4% paraformaldehyde for 30 min at ambient temperature. After three PBS washes, cells were permeabilized using 0.1% Triton X-100 for 10 min. Nonspecific binding was blocked with 5% bovine serum albumin for 2 h at ambient temperature. Cells were incubated with primary antibodies overnight at 4 °C. Following further PBS washes, cells were incubated with fluorophore-conjugated secondary antibodies for 1 h at room temperature, protected from light. Nuclei were counterstained with 4′,6-diamidino-2-phenylindole (DAPI) for 15 min before imaging. Fluorescence microscopy was performed using a Leica TCS SP8 laser-scanning confocal microscope (Leica Microsystems).

### Transcriptome analysis

PBMCs were treated with XM2020 or HuB2 (multiplicity of infection [MOI] =1) for 24 h (3 biological replicates in each group). RNAiso Plus (1 ml; TaKaRa) was added to each sample, and the total RNA was extracted, following the manufacturer’s instructions. The quantity and quality of the RNA samples were measured using a NanoPhotometer spectrophotometer (Implen). The integrity of each RNA sample was assessed with an Agilent 2100 Bioanalyzer (Agilent Technologies). Nine RNA samples were used to generate the cDNA libraries. The libraries were sequenced on an Illumina HiSeq 2500 sequencer (Illumina). All of the clean reads were aligned to the reference genome using STAR. Differential expression gene analysis of the two groups was performed using the DESeq2 R package (1.16.1). The RNA-seq data generated in this study have been deposited in the NCBI Sequence Read Archive (SRA) under BioProject accession number PRJNA1417722.

### Statistical analysis

The mean ± standard deviation (SD) of three biological replicates was used to display all quantitative results. Statistical comparisons between experimental groups were performed using two-tailed Student's t-tests in GraphPad Prism 8.4 (GraphPad Software). For all analyses, a *p*-value less than 0.05 was considered statistically significant.

## Data availability

The RNA-seq data generated in this study have been deposited in the NCBI Sequence Read Archive (SRA) under BioProject accession number PRJNA1417722.

## Supporting information

The following are the Supplementary data to this article: Supplementary information.docx.

## Conflict of interest

The authors declare that they have no conflicts of interest with the contents of this article.

## Figures and Tables

**Supporting Figure S1:**
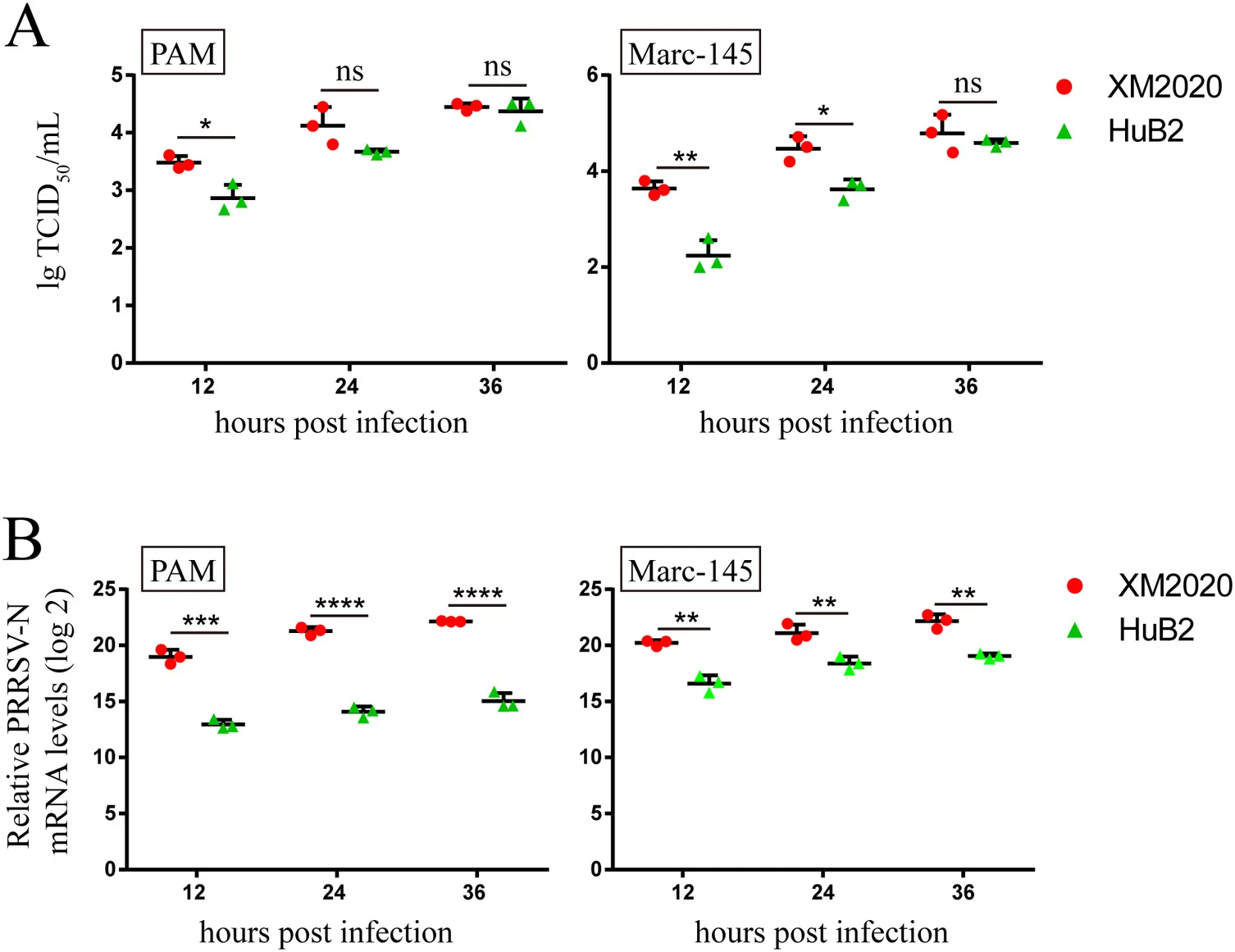


**Supporting Figure S2:**