# Supplementary material for: Protein M of NADC30-like PRRSV induces MARCH6-mediated K33-linked polyubiquitination of SLA-DRA
Source: J Biol Chem. 2026 Aug 25;302(10):113478. doi: 10.1016/j.jbc.2026.113478 (PMC13628661; doi:10.1016/j.jbc.2026.113478)
Supplement: Supplementary file 1 [file mmc2.docx]

**Figure legends:**

**S1 Fig. XM2020 demonstrated higher replication kinetics than HuB2 in vitro.**

(A-B) Growth Curves of XM2020 and HuB2 Strains. PAM and Marc-145 cells were infected with XM2020 or HuB2 (MOI=0.1). Samples were collected at the indicated times. Measure PRRSV N mRNA expression and viral titers in cell lysates and supernatants using TCID50 assay (A) and qPCR (B). Mean values ± SDs are shown (n = 3). Significance was assessed by two-tailed, unpaired Student’s t tests. ns, not significant; *P < 0.05, **P < 0.01, ***P < 0.001, ****p < 0.0001.

**S2 Fig. XM2020 downregulates the SLA-DR expression in XM2020-convalescent pigs’ PBMCs, which is independent of viral replication.**

(A-D) Verification of upregulated and downregulated genes in the RNA-seq via qPCR. PBMCs from XM2020-convalescent pigs were treated with XM2020 or HuB2 (MOI=1) for 24 h. The cell lysates were collected and subjected to qPCR to validate the differentially expressed genes from the RNA-seq analysis. (E) PBMCs from XM2020-convalescent pigs were treated with XM2020, and samples were collected at indicated times. The cell lysates were collected to analyze PRRSV N mRNA expression.
